# Mitral Incompetency—With Consequent Pulmonary Lesion

**Published:** 1895-10

**Authors:** S. Mills Fowler

**Affiliations:** Chicago, Ill.


### MITRAL INCOMPETENCY—WITH CONSEQUENT PULMONARY LESION.

S. Mills Fowler, M. D., Chicago, Ill.

### Case I.

Mrs. M. came under my care in December, 1892. She is a boarding-house keeper, aged fifty-seven years; married; mother of five children; the youngest being now about twenty-three years old; all of the children have been healthy; there is no history of tubercle or heart disease in her family on either side; her father died of what was supposed to be cancer of the stomach. This patient has had typhoid fever, malarial fever, and two years previous to coming under care sustained a severe attack of the then epidemic la grippe, from which she has never entirely recovered. She has taken on several occasions Calomel, and Quinine, until the characteristic drug effects were produced. For several years she had been under the professional care of a low-potency, alternating homœopath, who treated her during the attack of la grippe.

She is of quiet, rather cheerful disposition, and very hopeful, a mental condition so often met with in patients suffering from organic diseases, from which there is no hope of recovery. She is tall, spare, and angular, blue eyes, and brown hair, but slightly sprinkled with gray. For years she has had a cough, but more particularly since the la grippe experience. She has had several hemorrhages, some of which have been quite profuse. The cough is peculiar, it generally consists of two little hacks which are repeated every few seconds to a few minutes, and always dry. When there is any expectoration, it comes up with a little hemming and hawking. When there is any expectoration, it is quite free, of varying consistency from thin watery mucus to thick tenacious phlegm, but no pus. The hemorrhages come on suddenly, without warning, are of bright red blood, and coagulate readily. Then the expectoration will be blood-tinged for a day or two and may be somewhat frothy. The expectoration is never specked and streaked with blood as it is in bronchitis.

There is much emaciation and weakness. Night-sweats have been a prominent feature. At times some hectic. Some œdema about the eyes, and at times the feet and ankles are swollen.

From the foregoing one might be easily led to pronounce this a case of consumption or of chronic bronchitis, and such it always has been diagnosed by my professional predecessors in the case.

The interesting portion of the case here follows: We apply the "Methods of Physical Diagnosis," and try to learn the true nature and character of the affection.

*Inspection*, shows us a narrow, flat chest, the ribs, clavicles, and scapulæ, all standing out prominently. The respiratory movements are uniform, easy, and very nearly normal, the inferior-costal breathing movements on the right side are a little exaggerated, while the superior-costal movements on the left side are a little diminished. The heart's impulse is exaggerated and somewhat diffused. There is a little unnatural bulging of the cardiac region.

*Palpation*, shows that the heart action is not particularly forcible, and the impulse is diffused; tactile fremitus is about normal.

*Menstruation*, shows a chest expansion of two and one-half inches, a fair average for one of her form and build.

*Percussion*, shows a somewhat exaggerated resonance, but taking into account the thin walls and emaciated condition of this patient, are not indicative of serious lesions. There is an increased area of precordial dullness, which is distinctly abnormal and suggestive, if not quite diagnostic.

*Auscultation*, reveals, in the pulmonary areas, quite normal respiratory murmurs, as a rule, but at times there are present numerous râles, of all sorts, from the fine crepitant to the coarse bubbling and gurgling. In the cardiac area, however, we have a very pronounced systolic murmur, which has its point of maximum intensity at the apex, and can be distinctly heard in the axilla and at the back along the left side of the spine. This murmur somewhat masks the cardiac first sound. There is also an exaggeration of the pulmonary second sound.

Now the diagnosis is clear, and this is not consumption or bronchitis at all, but an endo-carditis with mitral insufficiency; a mitral regurgitation and consequent hypostatic congestion of the lungs; a functional disturbance of the lesser circulation.

Years ago Mrs. M. had enlargement of the heart; technically known as hypertrophy. Hers was hypertrophy with dilatation. Compensation was fairly maintained until the attack of la grippe made such serious inroads on her general health that the physical energies were no longer able to maintain it. She is now the victim of broken compensation, which is always sooner or later the sequel of hypertrophy with dilatation.

It is not reasonable to expect a radical cure in cases of this character, but we can do for them what has been done for this; by a careful selection of remedies we can make them comfortable and prolong lives of usefulness.

In these cases there is neither ulceration, tubercle, or foreign substance of any sort. The hemorrhage comes from an aneurismal varix, very like the bleeding of hemorrhoids from the rectum, and although dependent on a constitutional chronic miasm, there is not the danger in giving the deeper-acting antipsorics that there is in the other forms of chronic diseases which are characterized by the presence in the tissues of tubercle, the deposit of lime-salts, or other foreign substances.

Mrs. M. has taken Acon., Ars., Chin-off., Chin-sul., Lach., Lycop., Merc., Merc-dulc., Puls., Sil., Sul. in the higher potencies, as indicated, with very satisfactory results.

She is now, June 26th, 1894, in better health than at any time since the attack of la grippe in February, 1890.

This case illustrates just what Hahnemann teaches in Section III of *The Organon*, which in Wesselhœft's translation reads as follows:

"The physician should distinctly understand the following conditions: what is curable in diseases in general, and in each individual case in particular—that is, the recognition of disease (*indicatio*). He should clearly comprehend what is curative in drugs in general, and in each drug in particular—that is, *he should possess a perfect knowledge of medicinal powers.* He should be governed by distinct reasons, in order to insure recovery, by adapting what is curative in medicines to what he has recognized as undoubtedly morbid in the patient—that is to say, he should adapt it so that the case is met by a remedy well matched with regard to its kind of action (selection of the remedy, *indicatum*), its necessary preparation and quantity (proper dose), and the proper time of its repetition. Finally, when the physician knows in each case the obstacles in the way of recovery, and how to remove them, he is prepared to act thoroughly, and to the purpose, as a true master of the healing art."

I have underscored, and would further emphasize the sentence, "He should possess a perfect knowledge of medicinal powers."

I believe that Hahnemann recognized the danger in antipsoric treatment of tuberculosis, and diseases generally that are characterized by the presence in the tissues of foreign substances, and had it all in mind when he wrote this section of *The Organon.*

When the physican distinctly understands what is curable in diseases, clearly comprehends what is curative in drugs, and possesses a perfect knowledge of medicinal powers, then there will result from his labor a minimum of danger and a maximum of safety.

### Case II.—Puerperal Fever.

Mrs. R., aged about thirty-five. A strong, robust German woman. In her sixth confinement. I was hastily summoned about 8 o'clock on Wednesday morning in January last. About 3 A. M. she had a severe chill which lasted nearly an hour. I had been in the house but a few minutes; only long enough to find a temperature of 104°, and asked a few questions, when a Catholic priest who had been sent for arrived, and I, with all of the household except the patient and her new-born infant, were literally turned "out in the cold" for more than an hour. After this a discussion was held as to who should take charge of the case. The priest thought that the doctors could do nothing for her. He had performed the "last rites of the church," and that was all that could be done.

Finally I was again admitted to the sick-room, to do what I could.

About a week previously she had been confined under the tender (?) ministrations of a young allopath, assisted by a middle-aged eclectic. She had at the time of her labor been thoroughly ergotized, and then finally delivered with instruments.

Subsequent to delivery, she had been "douched" with clock-like regularity, by direction of the regular (?) physician every four hours, with hot medicated lotions of some sort.

The nurse reported that at times there had been a decided fetor to the discharges, but that she had kept it down with disinfectants.

At 11 A. M. her temperature was 106° F., and had risen at the rate of about a degree an hour from the termination of the chill. Pulse was 140, full, hard, and flowing (the opposite of bounding). She was bathed in perspiration, and though covered in blankets and a feather-bed, complained of feeling chilly. The abdomen was much distended and tympanitic. Nearly the whole body, the face, neck, and arms, was covered with a dark-red rash, somewhat resembling measles. Her mouth and throat was so dry that she could scarcely speak, yet she wanted no drink; was not thirsty. The lochia had been suppressed, and there was no milk in her breasts.

There was no doubt in my mind that there was some influence at work here more than the natural ones and recognized it as that of the Ergot. It seemed to me that this influence must be counteracted. For this purpose a single dose of Secal-cor.$^{cm}$, was given dry on the tongue.

The remedy unhesitatingly was Puls., which was given in solution every hour.

At 1 P. M. the temperature had not advanced, but remained at 106°.

At 2 P. M. it had fullen to 105°.

Placebo was then prepared and given every two hours.

At 8 P. M. the temperature was 102°, with a decided change for the better.

No more medicine was given, and the patient was convalescent the following Sunday, and has needed nothing since that I am aware of.

